# The cytotoxicity of polycationic iron oxide nanoparticles: Common endpoint assays and alternative approaches for improved understanding of cellular response mechanism

**DOI:** 10.1186/1477-3155-10-15

**Published:** 2012-04-17

**Authors:** Clare Hoskins, Alfred Cuschieri, Lijun Wang

**Affiliations:** 1 Institute for Medical Science and Technology (IMSaT), Wilson House, 1 Wurzburg Loan, University of Dundee, Dundee DD2 1FD, UK

**Keywords:** Magnetic nanoparticle, Cellular interaction, Cell membrane, Cytotoxicity, Cell viability assay, Atomic force microscopy, Zeta potential

## Abstract

**Background:**

Iron oxide magnetic nanoparticles (MNP's) have an increasing number of biomedical applications. As such in vitro characterisation is essential to ensure the bio-safety of these particles. Little is known on the cellular interaction or effect on membrane integrity upon exposure to these MNPs. Here we synthesised Fe_3_O_4 _and surface coated with poly(ethylenimine) (PEI) and poly(ethylene glycol) (PEG) to achieve particles of varying surface positive charges and used them as model MNP's to evaluate the relative utility and limitations of cellular assays commonly applied for nanotoxicity assessment. An alternative approach, atomic force microscopy (AFM), was explored for the analysis of membrane structure and cell morphology upon interacting with the MNPs. The particles were tested in vitro on human SH-SY5Y, MCF-7 and U937 cell lines for reactive oxygen species (ROS) production and lipid peroxidation (LPO), LDH leakage and their overall cytotoxic effect. These results were compared with AFM topography imaging carried out on fixed cell lines.

**Results:**

Successful particle synthesis and coating were characterised using FTIR, PCS, TEM and ICP. The particle size from TEM was 30 nm (−16.9 mV) which increased to 40 nm (+55.6 mV) upon coating with PEI and subsequently 50 nm (+31.2 mV) with PEG coating. Both particles showed excellent stability not only at neutral pH but also in acidic environment of pH 4.6 in the presence of sodium citrate. The higher surface charge MNP-PEI resulted in increased cytotoxic effect and ROS production on all cell lines compared with the MNP-PEI-PEG. In general the effect on the cell membrane integrity was observed only in SH-SY5Y and MCF-7 cells by MNP-PEI determined by LDH leakage and LPO production. AFM topography images showed consistently that both the highly charged MNP-PEI and the less charged MNP-PEI-PEG caused cell morphology changes possibly due to membrane disruption and cytoskeleton remodelling.

**Conclusions:**

Our findings indicate that common in vitro cell endpoint assays do not give detailed and complete information on cellular state and it is essential to explore novel approaches and carry out more in-depth studies to elucidate cellular response mechanism to magnetic nanoparticles.

## Background

Recently magnetic nanoparticles have become the focus of scientific interest due to their vast biomedical applications [1-3]. The solution instability and toxicity of iron oxide nanoparticles have been extensively studied and overcome by complete coating of the particles using materials such as silica [4], polymers [5], inorganic metals [6], bioactive molecules [7] etc. After coating the MNP core the acute toxicity experienced is attributed to the physicochemical properties of the particle surface [8]. Such properties include hydrodynamic radius [9], surface charge [10] and inherent toxicity of the coating materials [11]. However, little is known of the mechanism of cellular interaction and the long term stability of these particles in physiological conditions [12]. Cellular fate is dependent on cellular responses to acute toxicity, toxicity of degradation products and toxic effects due to nanoparticulate systems [3]. As such as more applications for magnetic nanoparticles are realised priority should be placed on the understanding of mechanisms of nanoparticle-cell interaction and cellular response that underline the toxicity of these particles.

It is presumed that after cellular uptake via endocytosis the clusters of iron oxide nanoparticles reside inside lysosomes [13] followed by degradation into iron ions via enzyme hydrolysis in the low pH environment [2]. It has been reported that the reactive oxygen species induced by transition metal particles can lead to lipid peroxidation [14,15]. Lipid peroxidation results in the disruption of the phospholipid bilayer membrane as a result of intracellular stresses from hydrogen peroxide production (Eqn. 1&2) [16,17]; this can also result in cell mortality [18].

(1) $$\left[{{\text{O}}_{2}}^{-}+\text{F}{\text{e}}^{3+}\to {\text{O}}_{2}+\text{F}{\text{e}}^{2+}\right]$$

(2) $$\left[2{{\text{O}}_{2}}^{-}+2{\text{H}}^{+}\to {\text{O}}_{2}+{\text{H}}_{\text{2}}{\text{O}}_{\text{2}}\right]$$

In 1987 Minotti and Aust investigated the requirement for iron (III) in the initiation of lipid peroxidation [19]. Their findings suggested that lipid peroxidation can only be initiated by the presence of both Fe^2+ ^and Fe^3+ ^as alone neither Fe^2+ ^nor Fe^3+ ^could promote peroxidation of the lipid membrane [19]. This finding suggests that lipid peroxidation will occur in cells with internalised Fe_3_O_4 _only if degradation of the particles occurs.

The oxidation of Fe^2+ ^by H_2_O_2 _believed to initiate redox cycling promoting the free radical production via the final step of the cycle, is known as Fenton's reaction (Eqn. 3) [2,19]. This free radical production causes intracellular stresses and can lead to cellular death [20,21]. Soenen et al. reported significantly increased free radical production in C17.2 neural progenitor, PC12 rat pheochromocytoma and human blood outgrowth cells incubated with clinically available Endorem^® ^and three other iron oxide nanoparticles (Resovist^®^, magnetoliposomes and very small iron oxide nanoparticles) [11]. Although the stress levels were significantly greater than control cells, Soenen et al. concluded that the contribution of nanoparticle-induced oxidative stress to the toxicity of these nanoparticles remained unclear as cells possess inherent defence systems in order to deal with varying oxidative stress levels [11].

(3) $$\left[{\text{H}}_{2}{\text{O}}_{2}+\text{F}{\text{e}}^{2+}\to \text{F}{\text{e}}^{3+}+\text{O}{\text{H}}^{-}+\text{O}{\text{H}}^{*}\right]$$

In order to obtain a comprehensive safety profile of iron oxide MNPs various studies should be carried out which measure different aspects of the cellular response [22]. Routine analysis for cytotoxicity of nanoparticles is largely based on methods established for hazard characterisation of chemicals or cytotoxic drugs, using assays such as the MTT (absorbance) or CellTiter Blue (fluorescence). As reported in several previous studies including our own [22-24], these commonly used endpoint assays which measure cellular enzyme activity frequently interact with nanoparticles and in our case, consistently over-estimated cell viability when validated with traditional Trypan blue counting [24]. Commonly cytotoxicity data are used to evaluate the cellular fate after exposure to magnetic nanoparticles; however these endpoint assays do not elucidate the cellular physiological state. Cells impermeable to Trypan blue are assumed to be viable and healthy; however, is this always the case and to what extent do these in vitro studies reflect in vivo conditions? Feridex is a dextran coated superparamagnetic iron oxide nanoparticle clinically administered in MRI imaging of patients [25]. Although FDA approved [25], Feridex still causes adverse reaction in patients [26]. These reactions can lead to hypotension, liver lesions, anaphylactic reaction which in severe cases can be lethal [26,27]. The reasons for inter-patient sensitivities are not well understood. In order for future novel metallic nanostructures to be safe for patient use we believe understanding cellular state in response to nanoparticle exposure is of utmost importance.

Atomic force microscopy (AFM) is an established characterisation technique used for topographic imaging especially in the physical sciences for materials such as polymers [28], microchips [29] etc. With advances in technology this powerful tool can now be applied to biological samples [30]. The ability to obtain topography images of cells allows for detailed cell morphology visualisation which before was unobtainable [31]. Cell membrane interaction with nanoparticles is a largely unknown area. Studies have shown that upon cellular incubation with nanoparticles nanosized pores develop in the cell membrane [32,33]. Vasir and colleagues reported the use of AFM to image nanoscale holes in the cell membrane after cellular exposure to copolymer poly(D,L-lactide-co-gylcolide) coated iron oxide nanoparticles [33]. The noticed nanosized 'pits' appearing in the cell membrane approximately 50 nm in depth and 170 nm in width at the surface. They postulated that this could be due to the restructuring of the membrane during the initial phase of endocytosis [33].

Here we will synthesise magnetic Fe_3_O_4 _nanoparticles and coat with poly(ethylenimine) (PEI) and subsequently poly(ethylene glycol) (PEG) giving rise to differing surface positive charges. PEI is a common polycation used frequently for coating magnetic nanoparticles, drug delivery and as a transfection agent [34]. PEG is a hydrophilic polymer used frequently in the coating of magnetic nanoparticles, it possess' many desirable qualities such as increased biocompatibility and 'stealth' properties leading to increased circulation times [34]. After surface coating we will carry out in vitro biocompatibility studies and explore the potential of AFM in elucidating nanoparticles-cell membrane interactions using three human cell lines including neuroblastoma (SH-SY5Y), breast cancer (MCF-7) and macrophage-like (differentiated U937) cells.

## Results

### Synthesis and characterisation of MNPS

**Figure 1:**
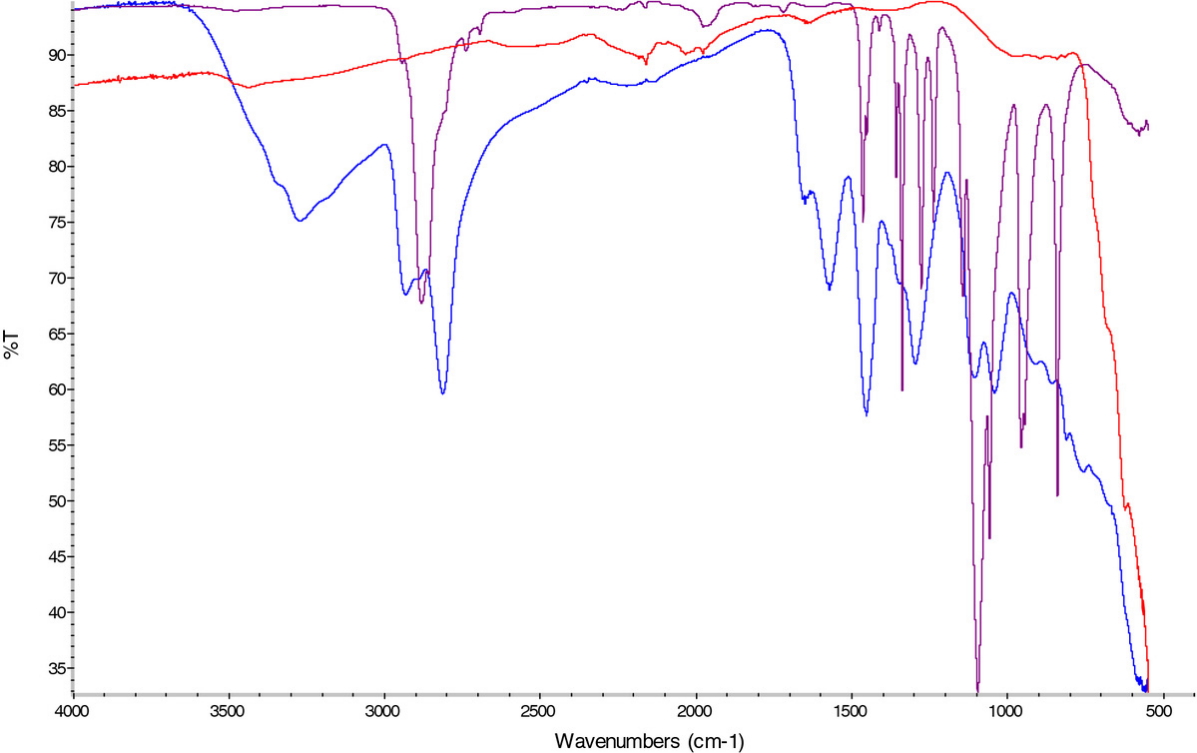
**FTIR spectra of freeze dried MNP (red), MNP-PEI (blue) and MNP-PEI-PEG (purple) carried out on a Nicolet IS5 with and ID5 diamond tip ATR attachment**. 64 scans were carried out for each sample.

**Figure 2:**
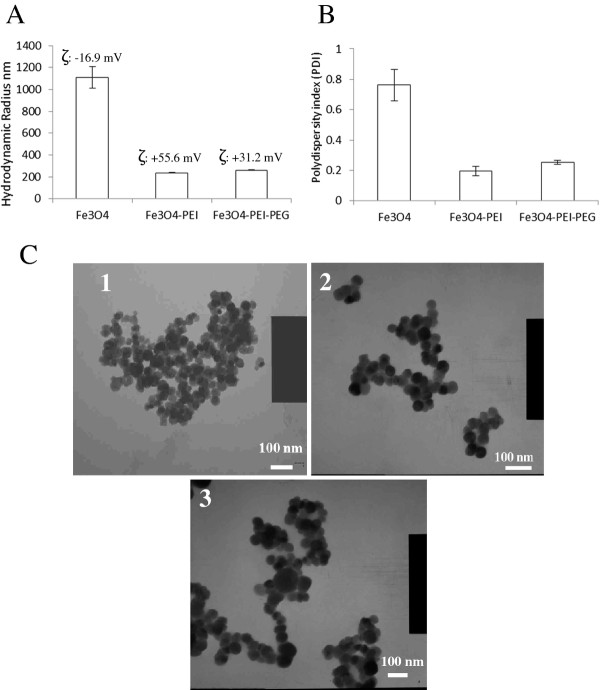
**Size estimations of MNPs analysed by A) Photon correlation spectroscopy showing the surface charge and B) Polydispersity index of particles measured at 1 mgmL^-1 ^in deionised water (n = 9 ± SD) and C) TEM images of 1) naked MNP, 2) MNP-PEI and 3) MNP-PEI-PEG**.

The particles were synthesized and coated with PEI and PEG. Fourier transform infrared spectroscopy (FTIR) analysis of the freeze dried particles confirmed the attachment of polymer backbone to the 'naked' particles with the presence of -NH peaks at 3300, 1700 & 1600 cm^-1 ^and a distinct C-N peak at 1000 cm^-1 ^(Figure 1). The broad peak observed at 3100 cm^-1 ^was due to free water which was still present in this hygroscopic polymer even after 8 h freeze drying. The distinction between MNP-PEI and its pegylated counterpart was made via the absence of the primary amine peaks at 3300, 1700 &1600 cm^-1 ^which were observed in the MNP-PEI sample. The alkyl peak at 2800 cm^-1 ^appeared more pronounced in the presence of the PEG moiety due to the nature of the polymer backbone. Additionally a small peak was observed at 3400 cm^-1 ^which was due to the C = O stretch of the bonds in the PEG moiety (Figure 1). Inductively coupled plasma (ICP) spectroscopy was used to deduce the concentration based on the total iron content of the MNPs. The 'naked' MNPs had a hydrodynamic radius of 1112 nm determined by photon correlation spectroscopy (Figure 2A). This large value indicated that aggregation of the individual particles had occurred due to their inherent magnetic properties, hence a large polydispersity index was observed (0.763) (Figure 2B). The TEM micrograph gave a more realistic representation of the MNP size which was approximately 30 nm (Figure 2C1). After PEI coating the concentration was 13.5 mgmL^-1 ^(93% yield) and 11.1 mgmL^-1 ^(82% yield) after subsequent pegylation. The polymer coated nanoparticles appeared more stable in solution and aggregation was reduced with the hydrodynamic radius for both MNP-PEI and MNP-PEI-PEG significantly reducing to 237 nm and 262 nm respectively (p < 0.005) (Figure 2A). The surface charge of the MNPs was determined by zeta potential measurement. The 'naked' MNP's possessed a negative surface charge (−16.9 mV) due to sulphate associations previously reported to result from the synthetic route [35] (Figure 2A). Addition of PEI increased the overall zeta potential measurement to +55.6 mV due to the positive charge on the amine groups of the polymer backbone; this measurement gives good indication that coating was successful. The zeta potential measurement for the pegylated particle was +31.2 mV. The decrease observed is attributed to the presence of -OH groups on the PEG coating as previously reported [24].

### Stability of coated MNPs

**Figure 3:**
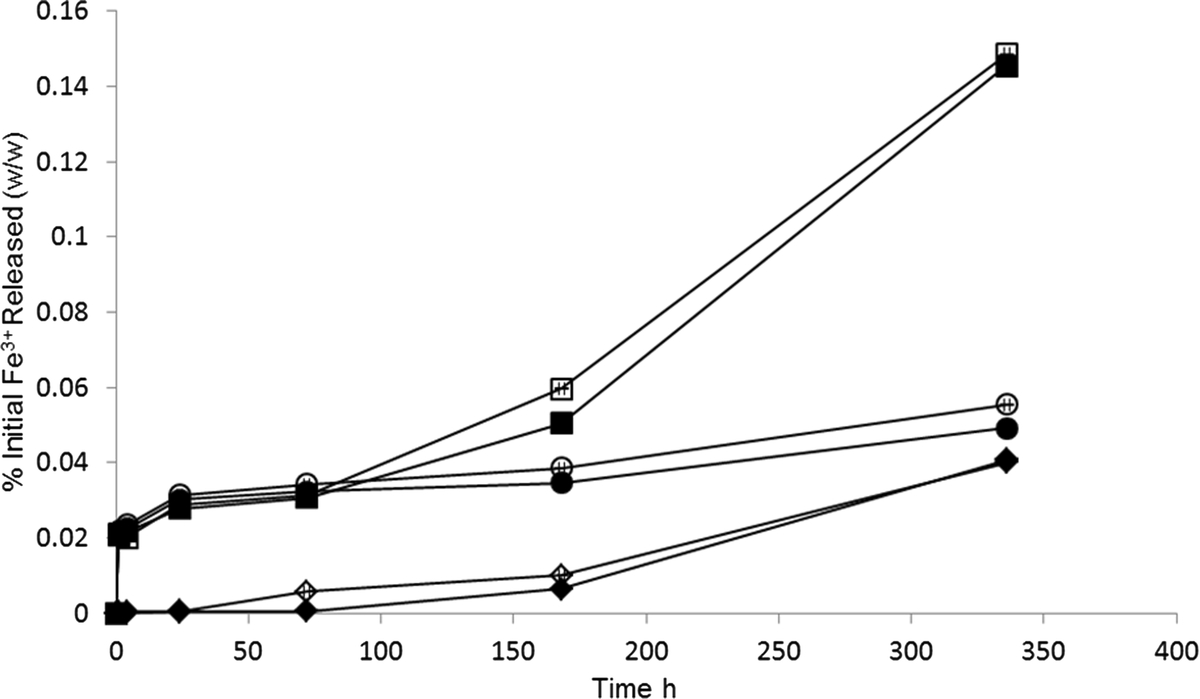
**Stability of 1)MNP-PEI (open marker) and 2) MNP-PEI-PEG (filled marker) in RPMI-1640 media of pH 7.2 (◇), pH 4.6 (○) and pH 4.6 containing sodium citrate (20 mM) (□). Study carried out using 2 mL, 100 μgmL^-1 ^MNPs under *sink *conditions with stirring over 2wks (n = 3 ± SD)**.

The stability of MNPs against degradation at pH's mimicking physiological (7.2) and intracellular (4.6 and 4.6 with 20 mM citrate ions) environments were determined over a 2 week period (Figure 3). The results were expressed as a percentage weight of the initial starting MNP iron weight (200 µg). At pH 7.2 a maximum of 0.04% of the initial iron concentration was observed in the media for the MNP-PEI and its pegylated counterpart. The release rate for both MNPs appeared to be consistent over the time period. At pH 4.6 particle degradation appeared to be greater. An initial burst release of iron (0.027%) was observed in the first 24 h followed by a slow incline over the duration to a maximum of 0.055% and 0.049% for MNP-PEI and MNP-PEI-PEG respectively. The increased degradation compared with at higher pH can be attributed to the iron core being slowly dissolved in acidic environments. Upon addition of citrate ions into pH 4.6 media the iron degradation increased significantly after 1 week (p < 0.005), and up to a maximum of 0.149% and 0.145% for MNP-PEI and MNP-PEI-PEG respectively, in two week time. Citrate ions possess the ability to chelate with the iron molecules leading to hydrolysis and resulting in particle degradation [36]. In general, the MNP-PEI appeared to degrade slightly more when compared to the MNP-PEI-PEG; however this was not significant (p > 0.005). Based on the amount of released iron ion, the stability of our synthesized MNP's is comparable with that of clinically approved iron oxide nanoparticles ferumoxides [25] as shown by a previous study [37].

### Cellular uptake of nanoparticles

The intracellular content of MNPs in SH-SY5Y, MCF-7 and U937 cells was determined by ICP after incubation for 1, 4, 24 and 72 h with 25 µgmL^-1 ^MNPs (Table 1). The MNP cellular uptake appeared to be time dependant up to 24 h for both the MNP-PEI and MNP-PEI-PEG for in all cell lines. After 24 h the cellular uptake appeared to plateau indicating that the rate of cellular uptake was greatest within this time. The U937 cells resulted in lower intracellular iron levels after 72 h compared with the SH-SY5Y and MCF-7 cells, this finding is interesting when considering the phagocytotic nature of these cells. However, the relatively smaller cell volume of the U937 cells may result in a lower maximum uptake compared with the larger SH-SY5Y and MCF-7 cells.

**Table 1:** Cellular uptake of polymer coated MNP in SH-SY5Y, MCF-7 and U973 cells at 25 µgmL^-1 ^over 72 h (n = 3 ± SD).

Particle	Incubation time h	Concentration of Fe^3+ ^uptake per cell, pg (± SE)		
		
		SH-SY5Y	MCF-7	U937
MNP-PEI	0	0.560 (0.017)	0.261 (0.004)	0.121 (0.087)
	
	1	16.205(0.867)	7.990 (3.581)	7.017 (0.377)
	
	4	14.426 (0.577)	14.257 (0.415)	11.640 (0.405)
	
	24	23.277 (0.506)	16.167 (1.258)	12.057 (1.315)
	
	72	25.580 (0.353)	19.847 (1.305)	13.803 (1.842)

MNP-PEI-PEG	0	0.560 (0.017)	0.261 (0.004)	0.121 (0.087)
	
	1	8.227 (0.523)	7.027 (0.424)	5.163 (0.484)
	
	4	9.997 (0.451)	9.963 (0.791)	7.353 (0.380)
	
	24	17.770 (1.462)	14.257 (0.297)	9.740 (0.986)
	
	72	18.700 (0.360)	16.593 (0.756)	9.627 (0.997)

### Cell viability by nanoparticle exposure

**Figure 4:**
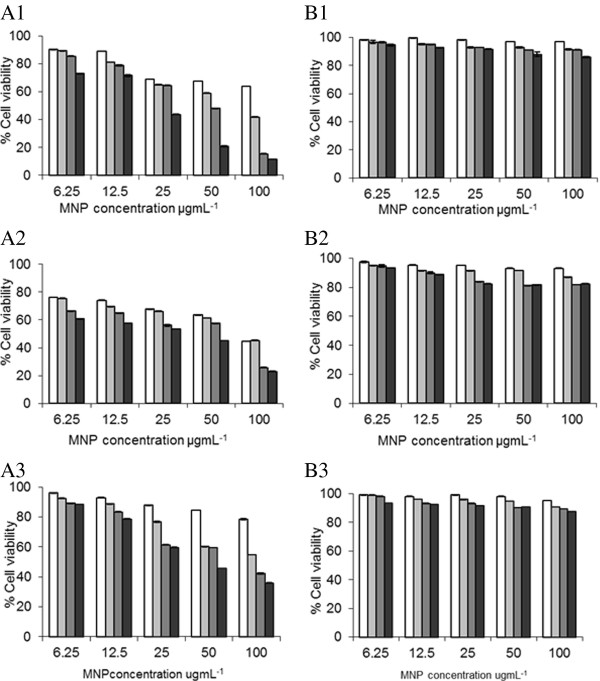
**Trypan blue exclusion assay using A) Fe3O4-PEI and B) Fe3o4-PEI-PEG on 1) SH-SY5Y, 2) MCF-7 and 3) U937 cells over □ 24, ■ 72, ■120 and ■168 h (n = 3 ± SD)**.

Viability in response to nanoparticles was evaluated by Trypan blue exclusion for cells exposed to nanoparticles over 7 days (Figure 4). After 24 h incubation with MNP-PEI up to 30%, 50% and 20% reduction in viability was observed in SH-SY5Y, MCF-7 and U937 cell lines respectively at 100 µgmL^-1^. However, the MNP-PEI-PEG experienced only a maximum of 5% viability reduction across the three cell lines. After 168 h the viability dropped to 10%, 20% and 30% for MNP-PEI incubated cells compared with around 90% for the MNP-PEI-PEG's in all cells.

### Cell membrane integrity after incubated with nanoparticles

The cell membrane integrity was determined via quantification of the LDH leakage from cells incubated with nanoparticles compared to control cells. In SH-SY5Y cells (Table 2) there was no increase from the basal values (10%) at all MNP-PEI concentrations during short incubation periods (1 h to 24 h) except at the highest concentration (100 µgmL^-1^) at 24 h. After 72 h incubation a significant increase (30%, p < 0.005) in LDH leakage was observed across the whole concentration range. The MCF-7 cells experienced an increase in LDH leakage after 24 and 72 h incubation with MNP-PEI (p < 0.005) (Additional file 1: Table S1). Similar to the SH-SY5Y cells a time dependant trend was observed irrespective of concentration. No increase from basal levels (6-7%) of LDH leakage was observed in the U937 macrophage-like cells. (Additional file 1: Table 2). In general the MNP-PEI-PEG did not cause LDH leakage in all three cell lines.

**Table 2:** Percentage cytotoxicity on cell membrane measured via LDH leakage using MNP-PEI and MNP-PEI-PEG on SH-SY5Y cells over 1, 4, 24 and 72 h (n = 3 ± SD)

Particle	Incubation time h			MNP concentration µgmL^-1^			
		
		0	6.25	12.5	25	50	100
MNP-PEI	1	10.29 (0.607)	12.89 (0.282)	12.15 (0.152)	12.16 (0.357)	12.66 (0.952)	14.42 (0.997)
	
	4	9.73 (0.014)	13.30 (1.052)	13.21 (1.192)	12.38(0.183)	12.16 (0.339)	12.35 (0.428)
	
	24	10.26 (0.014)	10.75 (0.080)	11.07 (0.344)	11.38 (0.021)	13.90 (0.132)	24.94 (0.789)*
	
	72	11.86 (0.288)	32.43 (2.366)*	35.51 (1.897)*	33.03 (2.103)*	30.86 (1.464)*	31.37 (0.788)*

MNP-PEI-PEG	1	10.44 (0.007)	10.43 (0.526)	9.95 (0.120)	9.85 (0.010)	9.80 (0.288)	9.93 (0.087)
	
	4	10.52 (1.539)	11.61 (0.616)	11.66 (0.158)	11.62 (0.350)	11.71 (0.102)	11.63 (0.484)
	
	24	10.35 (1.531)	9.02 (0.027)	8.67 (0.153)	8.84 (0.072)	9.00 (0.024)	9.50 (0.295)
	
	72	11.13 (0.242)	11.29 (0.591)	11.17 (0.443)	12.11 (2.964)	10.69 (0.254)	12.98 (0.000)

* Denotes a significant increase from basal levels (p < 0.05).

### Cellular oxidative stress measured by reactive oxygen species (ROS) production and lipid peroxidation

The ROS assay determines the intracellular stress levels due to the production of free radical oxygen species. For all three cell types tested the MNP-PEI caused significant increase in free radical production. In SH-SY5Y cells (Table 3) the PEI coated particles resulted in up to 20% increased stress levels compared with the control cells. The MNP-PEI-PEG did not appear to increase the oxidative stress levels in line with our previous findings [24]. The MCF-7 cells (Additional file 1: Table S3) appeared to experience much larger stress levels with up to 54% increase compared to the control cells when incubated with MNP-PEI. Again the pegylated particle did not appear to cause any stress due to free radical production. Similar to the SH-SY5Y cells, the U937 cells (Additional file 1: Table S4) experienced up to 10% elevated stress levels due to the MNP-PEI with no significant increase upon incubation with MNP-PEI-PEG. Hydrogen peroxide generation can occur inside cells as a secondary product from reactive oxygen species. The presence of these species can initiate stress to the cell membrane in the form of lipid peroxidation [19]. The results from the TBARS assay in SH-SY5Y cells can be seen in Table 3. These indicated that the MNP-PEI and MNP-PEI-PEG did not appear to result in any significant induction of lipid peroxidation in all three cell lines (MCF-7 Additional file 1: Table S3 and U937 Additional file 1: Table S4) tested when compared to control cells.

**Table 3:** ROS (% of control cell) and LPO induction by MNPs in SH-SY5Y cells incubated with 25 µgmL^-^^1 ^for 1, 4, 24 and 72 h (n = 3 ± SD).

Particle	Incubation time h	ROS Assay	LPO Assay
		
		% DCF fluorescence	MDA nM/mg protein (Control cells: 2.620 ± 0.225)
MNP-PEI	1	99.00 (5.568)	2.702 (0.015)*
	
	4	115.67 (5.033)*	2.567 (0.188)
	
	24	121.67 (7.371)*	2.667 (0.321)
	
	72	113.67 (4.509)*	2.638 (0.157)

MNP-PEI-PEG	1	97.33 (6.658)	2.282 (0.341)
	
	4	101.00 (5.292)	2.383 (0.018)
	
	24	103.33 (3.512)	2.383 (0.299)
	
	72	102.00 (2.000)	2.651 (0.107)

* Denotes a significant increase from basal levels (p < 0.05).

### AFM topography imaging of MNP - cellular interactions and cell membrane roughness analysis

**Figure 5:**
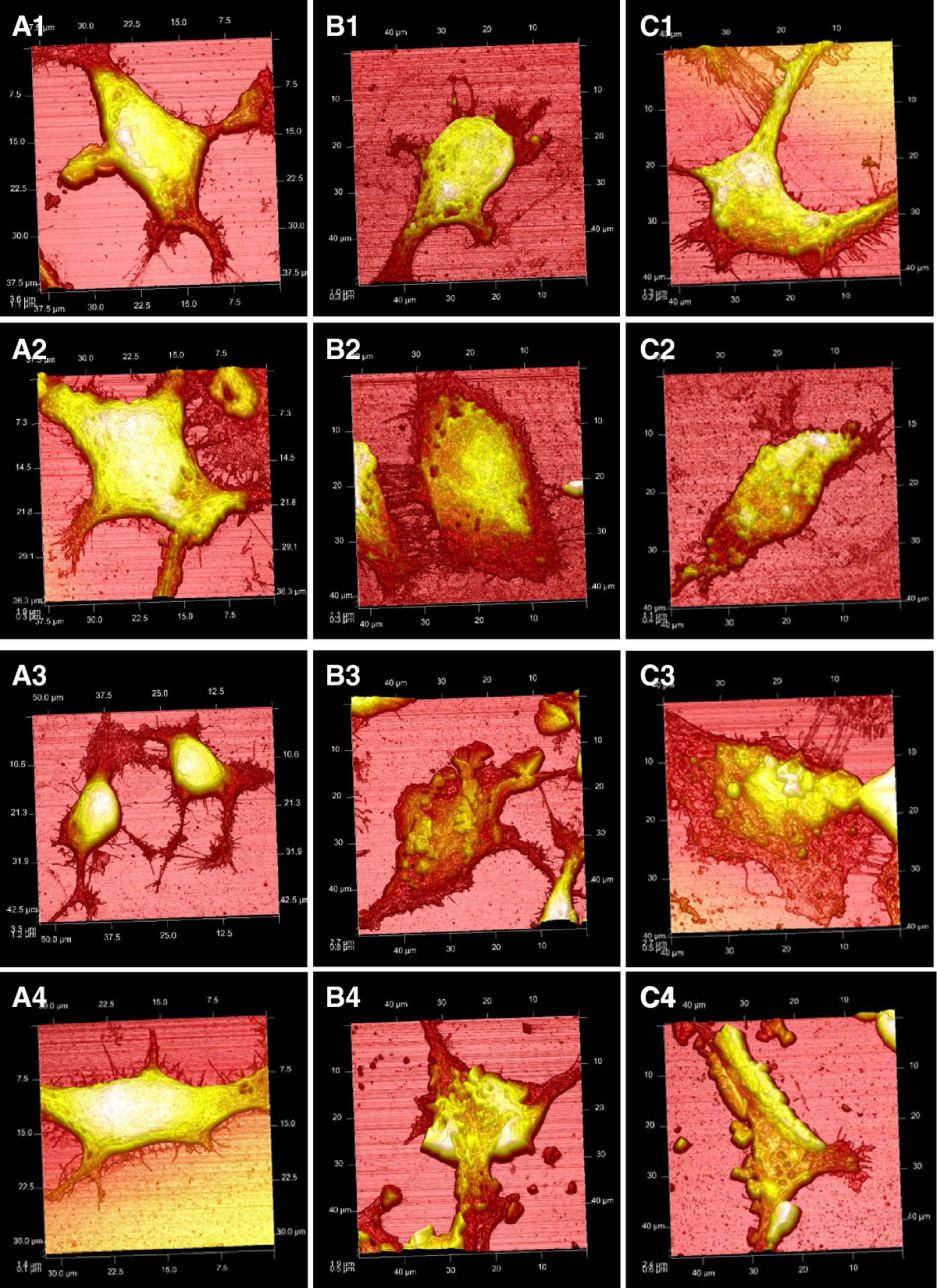
**AFM topography images of SH-SY5Y cells. A) control cells without MNPs, B) cells incubated with 25 µgmL^-1 ^MNP-PEI and C) MNP-PEI-PEG over 1) 1 h, 2) 4 h, 3) 24 h and 4) 72 h**. Cells were fixed after incubation and AFM imaging was performed in air using a RTESPA tip of spring constant 40 N/m, carrying out 896 scans/line at a scan rate of 0.32 Hz and 1.102 V amplitude.

In order to determine whether the cellular assays provided a good indication of the overall physiological state of the cell AFM topography images were obtained. Figure 5 shows the topography images for fixed SH-SY5Y cells, only one cell is shown for each time point (1, 4, 24, 72 h); however in practice extensive cellular images were obtained. These cells are shown as a representative of the overall cell state. The control cells (Figure 1, 2, 3, 4) appeared smooth and well formed with definite cell boundaries. This appearance was consistent throughout all the incubation times (1-72 h). After addition of the positively charged PEI-coated particles the cells appeared increasingly flattened and less structured. After 24 h incubation with MNP-PEI the cell morphology appeared to have completely changed. Interestingly, upon incubation with the MNP-PEI-PEG a similar trend was observed. Cell flattening had begun after only 4 h incubation with the particles andafter 72 h more dramatic change in cell surface topography was observed. A similar phenomenon was observed when both MNP-PEI and MNP-PEI-PEG were incubated with MCF-7 cells (Additional file 1: S1, Figure 1). With U937 the cell deformation appeared to be much smaller than that of SH-SY5Y and MCF cells (Additional file 1: S1, Figure 2). In all cells the MNP exposure appeared to cause small pits in the cell membrane resulting in a rougher cell surface. These results of morphological alteration represent a different pattern of cellular responses to iron oxide nanoparticles. These findings were in agreement in part with the cell membrane integrity measured via LDH leakage where a time dependant membrane disruption by MNP-PEI was observed and that MNP-PEI did not caused significant cell membrane damage in U973 cells (Table 2, Additional file 1: Table S1 and S2). These data also partially matched the level of the cellular oxidative stress in terms of ROS production by MNP-PEI (Table 3, Additional file 1: Table S3 and S4) where the increase in ROS reached its peak after 24 h incubation of MNP-PEI. As MNP-PEI-PEG were shown to induce neither cell membrane disruption nor cellular oxidative stress in all cells tested (Table 2, 3, Additional file 1: Tables S1, S2, S3, S4), the relationship between cell morphological change due to the interaction between MNPs and cells and measured cell parameters such as membrane integrity and oxidative stress are yet to be established. It should be noted that these traditional assays are endpoint measurements (and Trypan blue) and do not give an indication on the cellular dynamic physiology state. Perhaps AFM can be used as an important tool for assessing the biological effect these metallic nanostructures have in vitro which cannot be elicited by standard cell biological techniques.

**Figure 6:**
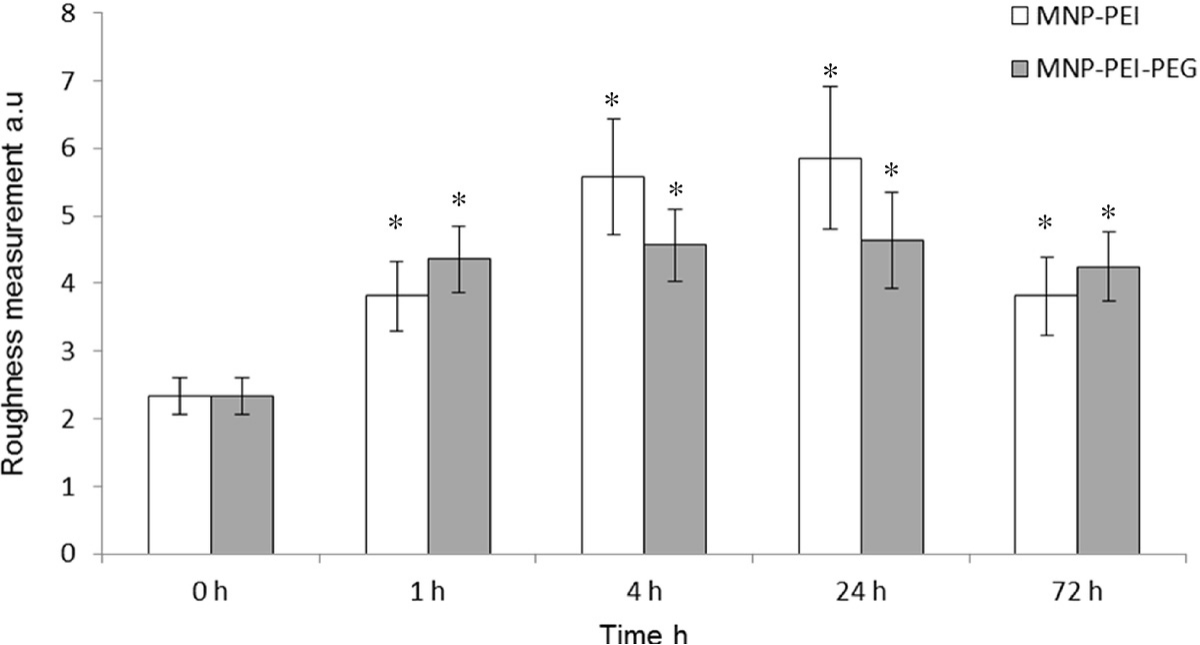
**Roughness analysis carried out on fixed SH-SY5Y cells of AFM topography images and analysed using NanoScope Analysis software (n = 3 ± SE)**. * Denotes a significant increase compared to control cells (p < 0.05).

The cell surface roughness analysis data on SH-SY5Y cells (Figure 6) further indicated that the cell membrane was affected by nanoparticle incubation [33]. These data are relative to control cells and serve only as a guide to describe the whole cellular state. In SH-SY5Y cells incubated with MNP-PEI the membrane roughness had virtually doubled after only 1 h. After 24 h incubation a 2.2-fold increase had occurred, however, after 72 h the roughness appeared to decrease slightly. These observations correlate with the level of MNP-PEI-induced ROS in which the largest increase was observed at 24 h incubation (Table 3, Additional file 1: Table S3, S4). This could also be a result of flattening of cells or adaptation of the cellular cytoskeleton in response to nanoparticle encounter. The decreased cell roughness with time was however not significant (p > 0.05). Similar to the MNP-PEI the cells that were incubated with MNP-PEI-PEG experienced an increase in membrane roughness over the duration of incubation. A decrease in membrane roughness compared to the 24 h values (p > 0.05) occurred after 72 h consistent with the MNP-PEI.

## Discussion

In this study we successfully synthesised magnetic iron oxide nanoparticles. The nanoparticles appeared to be mono-dispersed and around 30 nm in size (Figure 2C1). Polymer coating was achieved with both PEI and PEG which was confirmed with FTIR and zeta potential measurement. The MNPs appeared to be stable in conditions mimicking cellular pH up to 2 weeks with less than 0.15% of iron being released (Figure 3).

Increased cellular uptake was observed in the MNP-PEI compared with MNP-PEI-PEG which was attributed to the higher positive surface charge (+55.6 mV) attracting to the negative cell membrane and enhancing endocytosis [35]. The cell viability data (Figure 4) showed clearly that after pegylation of the MNP-PEI the cytotoxicity was significantly reduced (p < 0.005) in line with our previous findings [24]. The primary amines on the surface of the MNP-PEI give rise to the large positive surface charge (55.6 mV) and have previously been reported to cause a cytotoxic effect [38]. The reduction in cytotoxicity observed in the pegylated particles arises due to the decreased surface charge [38] and 'stealth' properties [34] on the particle surface. The highly charged MNP-PEI showed a concentration independent and time dependent effect on the LDH leakage from the SH-SY5Y and MCF-7 cells (Table 2 and Additional file 1: Table S1 respectively); this trend was not observed with the MNP-PEI-PEG with reduced surface charge where no deviation from the basal level was evident. The concept of Trypan blue exclusion and LDH leakage is similar however, the exact mechanism and molecular cut-off points for each molecule to pass the cell membrane is unknown. Perhaps this can explain the contrasting LDH leakage results where the cytotoxic effect is concentration independent compared with the Trypan blue exclusion.

In line with our previous study the MNP-PEI significantly (p < 0.005) increased ROS production resulting in cellular stress. After pegylation the stealth quality of the MNPs resulted in free radical production consistent with the control cells (Table 3) [24]. This result coupled with the stability data indicated that the free radical increase with MNP-PEI was likely to be caused by the increased positive charge on the polymer backbone and hence possible disruption of endosomal organelles [39] and not from the release of iron in the cytoplasm. Both MNPs showed no significant increase in lipid peroxidation (Table 3) suggesting that lipid peroxidation is not the major cause of cytotoxicity or other aspect of lipid oxidative stress not measured in this study was involved.

AFM topography imaging on fixed cells consistently showed that cellular morphology was dramatically altered after incubation with the MNPs (Figure 5 and Additional file 1: Figure 1). The greater cell topographical change in the SH-SY5Y cells perhaps could be attributed to the greater concentration of intracellular nanoparticles (Table 1). The U937 cells showed the smallest membrane structure change possibly due to their specialised functionality as phagocytic and scavenger cells and thus having a stronger cell defence capacity, as evidenced also by virtually no LDH leakage and very small increase in ROS production by MNP-PEI (Additional file 1: Table S2 and S4). The cell morphological observation by AFM in the SH-SY5Y and MCF7 cells was also in partial agreement with the level of the cellular oxidative stress (Table 3, Additional file 1: Table S3) where the increase in ROS reached its peak after 24 h incubation of MNP-PEI. Therefore, the link between cellular oxidative stress and cell morphological or other physical properties which could aid in improving our understanding of MNP toxicity and establishing more reliable methodology in toxicity evaluation, merits further investigation. Surprisingly, cells incubated with the lower charged MNP-PEI-PEG responded to nanoparticle exposure in a similar manner to the MNP-PEI, although no corresponding increase in LDH leakage and oxidative stress was found. The real nature of cell membrane morphology change during contact with nanostructures may be accredited to endocytosis [33] as well as possible membrane disruption by potential quantum mechanical effect and other nano-activities. Dissection of endocytosis-specific and "nano"-specific mechanisms underlying the change in cell membrane topography is currently undergoing in our lab.

As different aspects of MNP toxicity could contribute to their overall biological effect [22], the endpoint cytotoxicity, as judged in this study by Trypan blue exclusion, could be attributed to a complex combination of various factors, oxidative stress and cell membrane disruption being one of them. This is particularly important in the consideration of cell type-dependent responses, such as epithelial versus phagocytic immune cells, as immune cells (human macrophage-like U973 cells), could produce significant amount of cytokines in response to nanoparticles which in turn would greatly enhance the toxicity of nanoparticles under static cell culture conditions [40]. This may partially explain the negative membrane disruption and very small oxidative stress response and yet comparable (to the SH-SY5Y and MCF7 cell) overall cytotoxicity by MNP-PEI in U937 cells (Additional file 1: Table S2 and S4, and Figure 4).

## Conclusion

Our data indicates that the kinetics in cell morphology change resulting from iron oxide nanoparticle exposure may reflect a different aspect of cellular stress compared to those measured by conventional endpoint cell toxicity assays. As such we propose that these commonly used endpoint assays should not be used solely in determination of the safety profile of novel nanomaterials. In order to fully understand these observations more work needs to be carried out with regard to the cell membrane property and reorganisation of cytoskeletal system and alteration of other cellular properties in response to nanoparticles.

## Methods

All chemicals were purchased from Sigma-Aldrich unless otherwise stated.

### Synthesis of Fe_3_0_4 _nanoparticles

The synthesis was based on the established protocol of oxidative hydrolysis, i.e., the precipitation of an iron salt (FeSO_4_) in basic media (NaOH) with a mild oxidant [41]. In brief, nitrogen was bubbled through a solution of sodium hydroxide (0.1 M) and potassium nitrate (0.1 M) dissolved in deionised water at 90°C for 1 h. Iron sulphate (0.03 M) dissolved in sulphuric acid (0.01 M) was added to the reaction and the mixture was stirred for 24 h at 90°C under nitrogen. After this time the reaction was rapidly cooled on ice and the particles were washed X6 deionised water and magnetically separated from solution. The resultant particles were re-suspended in water and stored at 4°C.

### Coating and characterisation of MNPs

Iron oxide MNPS (2 mL) were sonicated in poly(ethylenimine) solution (5 mgmL^-1^) for 2 h. The particles were then washed X6 with deionised water and magnetically separated from solution. The MNP-PEI's were resuspended in 10 mL deionised water and stored at 4°C. MNP-PEI were added to 0.08 M sodium tetraborate followed by addition of methoxypolyethylene glycol p-nitrophenyl carbonate (mPEG, MW 5000) (20 mg) with stirring for 3 h at 22°C in the absence of light. The resultant solution was washed with deionised water and the MNP-PEI-PEG's eluted from solution using a high powered magnet. The MNP-PEI-PEG's were resuspended in deionised water and stored at 4°C. Freeze dried particles were run on the FTIR (Nicolet IS5 & ID5 ATR attachment, Thermo Scientific, UK) to determine whether polymer coating was successful. Nanoparticle concentration was determined using ICP analysis (Optima 7000 V DV, Perkin Elmer, UK). The particles were dispersed in deionised water and sonicated for 10 min before all measurements. Hydrodynamic diameters, polydispersity index and zeta potential measurements were carried out using a photon correlation spectrometer (Zetasizer Nano-ZS, Malvern Instruments, UK). All measurements were conducted in triplicate at 25°C and an average value was determined. Prior to zeta potential analysis standard control samples were run on the instrument.

### Degradation stability of coated MNPs

The stability of MNPs was evaluated based on established method [37] in RPMI-1640 media (Invitrogen, UK) with pH's representative of physiological (7.2) and intracellular (4.6) environments. Sodium citrate (20 mM) was added in pH 4.6 media to further mimic endosomal conditions. MNP solutions (2 mL, 100 µgmL^-1^) were placed inside dialysis membrane with molecular cut-off 12-14 KDa. The dialysis tubes were placed inside large conical flasks and stirred in 200 mL of appropriate media under 'sink' conditions. At 1,4,72,168 and 336 h a sample of media was removed (500 µL) and replaced with equal volume fresh media of similar pH. Sample media (100 µL) was added to 900 µL deionised water in an eppendorf tube. To each sample (1 mL), 4.95 mM bathophenanthroline disulfonic acid (40 µL) was added. The absorbance was measured after 90 s incubation at 535 nm (Techan M200 microplate reader). The samples were finally incubated with 100 mM ascorbate solution for 8 min before a final absorbance measurement was conducted at 535 nm. The final absorbance value was calculated as the positive difference between the initial reading subtracted from the final reading. The concentration of free Fe^3+ ^was calculated with respect to a standard curve (R^2 ^= 0.9943). The total free Fe3^+ ^was calculated as a percentage (w/w) in respect to the starting amount.

### Cellular uptake of nanoparticles measured by inductively coupled plasma (ICP)

SH-SY5Y, MCF-7 and U937* cells (ATCC, USA) seeded in 6-well plates and incubated with MNPs (25 µgmL^-1^) for 1,4, 24 and 72 h. The cells were washed X3 with PBS, trypsinised (Invitrogen, UK) and re-suspended in medium (Invitrogen, UK). The cell number was counted using a Countess™ Automated Cell Counter (Invitrogen, UK) and cells were placed in eppendorf tubes (1 × 10^6 ^cells/tube). The cell suspensions were centrifuged at 800 rpm for 5 min and the supernatant discarded. Concentrated hydrochloric acid (100 µL) was added to the cells and the tubes were incubated at 90°C for 0.5 h. The samples were cooled and centrifuged at 1500 rpm for 10 min. The supernatant was diluted with deionised water and run on the ICP (Optima 7000 V DV, Perkin Elmer, UK). A calibration was carried out using iron standard solutions 0.05 - 10 ugmL^-1 ^(R = 0.9999). A control sample of deionised water was also run.

*Differentiated U937 cells were used for all subsequent experiments to represent human macrophage-like cell conditions. Cells were differentiated by incubating cells with 10 nM Phorbol 12-myristate 13-acetate (TPA) for 3 days, followed by 1 day with fresh media prior to all experiments.

### Cell viability assay

Cell viability was determined using Trypan blue exclusion (Invitrogen, UK). Briefly SH-SY5Y, MCF-7 and U937 cells were seeded in a 12 well plate and incubated for 24 h at 37°C with 5% CO_2_. The cells were treated with increasing concentrations of MNP solutions (6.25 - 100 µgmL^-1^) and incubated for 24, 72, 120 and 168 h. The cells were washed with PBS x3 and trypsinised. Trypan blue was added to 100 µL cell suspension in equal volume and incubated for 5 min at room temperature. The viable cells were counted. Values of viability of treated cells were expressed as percentage of that from corresponding control cells. All experiments were repeated at least three times.

### Cell membrane integrity assay

Membrane integrity was measured via measurement of lactate dehydrogenase (LDH) leakage (Promega, UK) from SH-SY5Y, MCF-7 and U937 cells. Cells were seeded into a 96-well plate (10,000/well) and incubated for 24 h. The medium was replaced with increasing magnetic nanoparticles concentrations (6.25 - 100 µgmL^-1^). The plates where incubated for 1, 4, 24 and 72 h. Lysis buffer (2 µL) was added to positive control wells and the plate was centrifuged at 1500 rpm for 10 min at 37°C. The supernatant (50 µL) was then placed into a new plate and equal volume of membrane integrity assay reagent was added. The plates were incubated for 10 min at 37°C protected from light. 25 µL stop reagent was then added to the wells and the fluorescence of the samples was measured at 560 nm (excitation) and 590 nm (emission) on a Techan M200 microplate reader. The percentage of cytotoxicity in respect to the positive control wells was calculated whereby the lysed cells were assumed to have 100% LDH release.

### Reactive oxygen species (ROS) assay

SH-SY5Y, MCF-7 and U937 cells were seeded into a 96-well plate (10, 000/well) and incubated for 24 h. Cells were incubated with increasing MNP concentrations (6.25 - 100 µgmL^-1^) for 1, 4, 24 and 72 hrs. The cells were washed 3X with PBS and incubated for 1 h with 100 µM carboxy-H_2_DCFDA (Invitrogen, UK) in PBS at 37°C protected from light. The cells were washed 3X with PBS and incubated with serum free medium (100 µL) for 0.5 h. The medium was removed and replaced with PBS. The fluorescence intensity of the samples was measured at 560 nm (excitation) and 590 nm (emission) on a Techan M200 microplate reader. The percentage of DCF fluorescence was calculated in respect to control cells assumed to be 100%.

### Lipid peroxidation measurement by thiobarbituric acid reactive substance (TBARS) assay

SH-SY5Y, MCF-7 and U937 cells were seeded into a 6-well plate and incubated for 24 h. The medium was replaced with increasing MNP concentrations (6.25 - 100 µgmL^-1^) and cells were incubated for 1, 4, 24 and 72 h. The cells were washed 3X with PBS and trypsinised. The cells were resuspended in 0.5 mL PBS containing 0.05% butylated hydroxytoluene. The cell suspensions were sonicated for 5 s 3X at 40 V and kept on ice. Malondialdehyde bis(dimethyl acetal) (MDA) standard solutions (0-5 µM) were prepared and 100 µL of samples or standards were added to Eppendorf tubes. Sodium dodecyl sulphate (SDS) (100 µL, 2%) was added and the tubes were incubated for 5 min at room temperature. Thiobarbituric acid (250 µL) was added to the eppendorf tubes before incubation at 95°C for 1 h. The samples were cooled on ice and centrifuged at 3000 rpm for 15 min at 4°C. The supernatant was pipetted into the wells of a 96 well plate and fluorescent measurements were taken at 530 nm (excitation) and 550 nm (emission). The results were calculated as nmol of MDA/mg of cellular protein.

Protein content was determined by addition of 100 µL sample to 3 mL bradford reagent. The samples were mixed well at room temperature for 5 min and absorbance was measured at 595 nm. The absorbance values were compared to a calibration curve carried out using bovine serum albumin and the protein concentration was determined.

### AFM topography imaging of MNP - cellular interactions

SH-SY5Y, MCF-7 and U937 cells were seeded in 6-well plates containing collagen coated glass coverslips (SH-SY5Y cells used non-coated coverslips). Cells were incubated for 24 h at 37°C and 5% CO_2_. MNPs (25 µgmL^-1^) were added to the cells and further incubated for 1,4,24 and 72 h. Cells were washed X3 with PBS and fixed with 2.5% gluteraldehyde in PBS for 10 min. Fixed cells were washed X3 with deionised water and mounted on glass slides. Cell topography imaging was carried out using BioScope Catalyst AFM (Bruker, Germany) with ScanAsyst Adaptive Mode. Cell membrane topography was imaged using an RTESPA tip of spring constant 40 N/m, carrying out 896 scans/line at a scan rate of 0.32 Hz and 1.102 V amplitude. At least three cells were imaged to give a fair representation of each sample condition.

### Cell membrane roughness analysis

Cell membrane roughness was measured on the topography images using Nanoscope Analysis software (Bruker, Germany). Small areas (870 × 870 nm) were chosen at random on ten areas of each cell and their membrane roughness determined. An average was calculated from a total of thirty areas from three cells.

## Competing interests

The authors declare that they have no competing interests.

## Authors' contributions

CH carried out coating, characterisation, cell and AFM experiments, and wrote the paper. LY supervised the work and corrected the manuscript. AC was a scientific advisor and edited the manuscript. All authors read and approved the final manuscript.

## Supplementary Material

Additional file 1 **Supplementary data**. Figures 1 and 2 and Tables S1-S4. Click here for file
