# Supplementary material for: The cytotoxicity of polycationic iron oxide nanoparticles: Common endpoint assays and alternative approaches for improved understanding of cellular response mechanism
Source: J Nanobiotechnology. 2012 Apr 17;10:15. doi: 10.1186/1477-3155-10-15 (PMC3384250; doi:10.1186/1477-3155-10-15)
Supplement: Additional file 1 — Supplementary data. Figures 1 and 2 and Tables S1-S4. [file 1477-3155-10-15-S1.DOC]

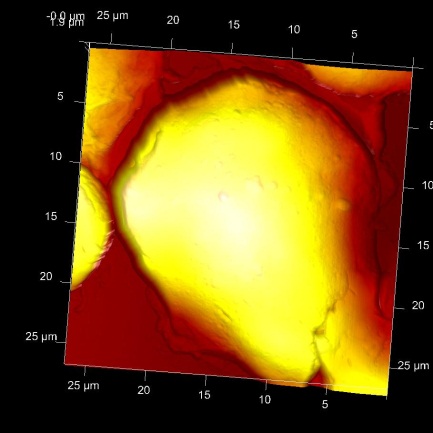

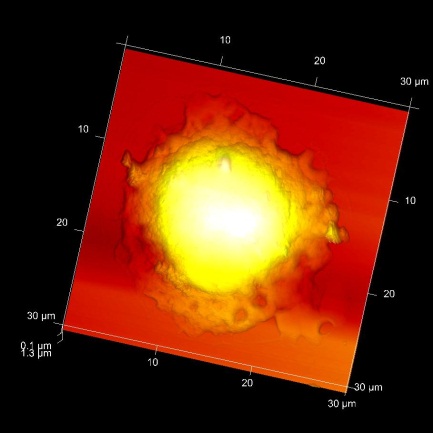

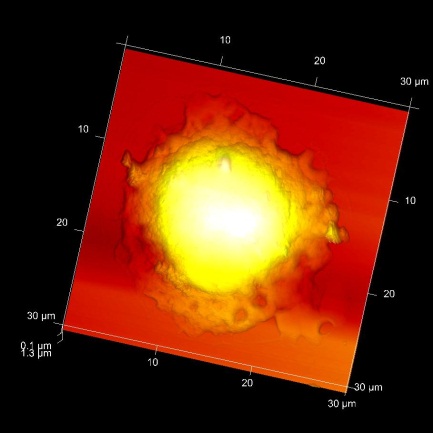


**C1**

**B1**

**A1**


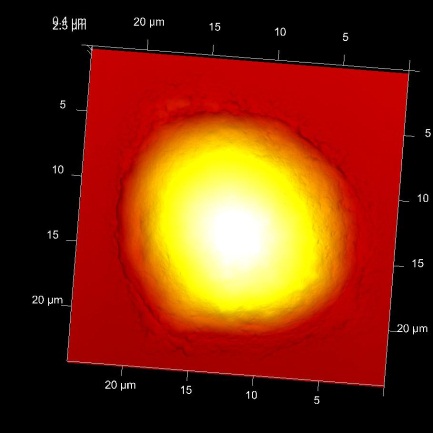

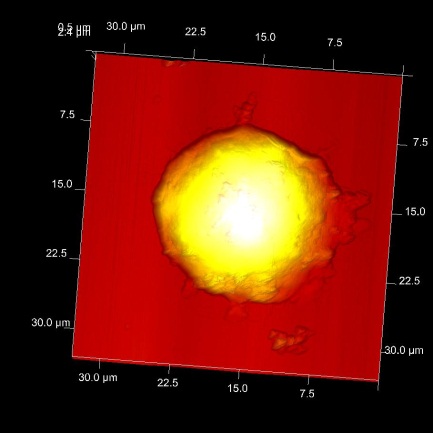

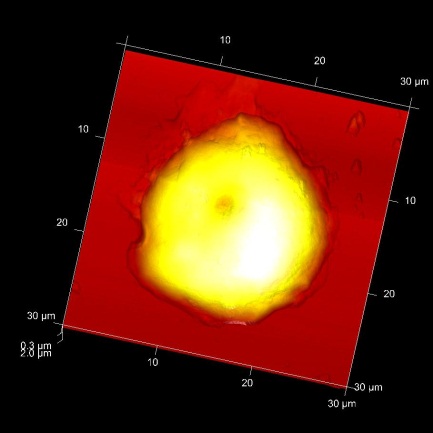


**A2**

**B2**

**C2**


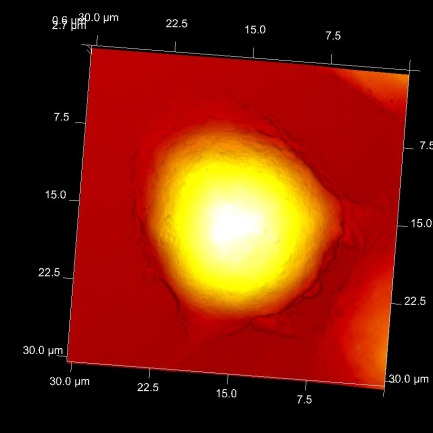

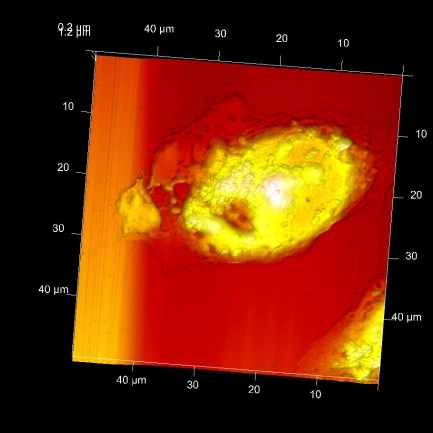

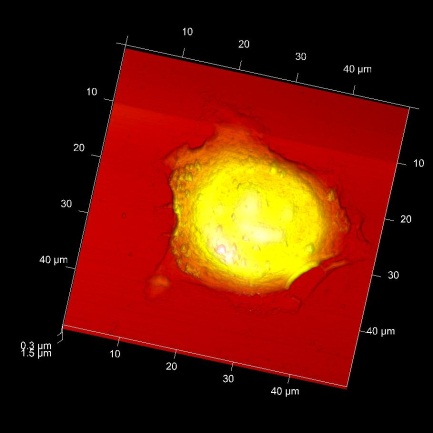


**A3**

**B3**

**C3**


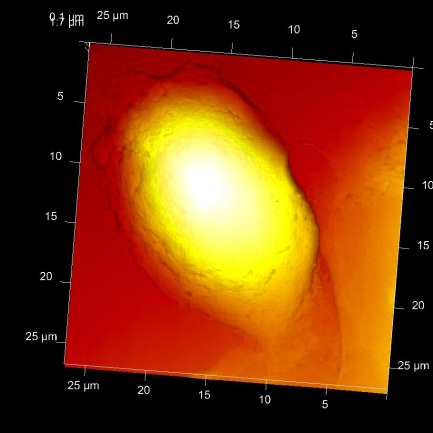

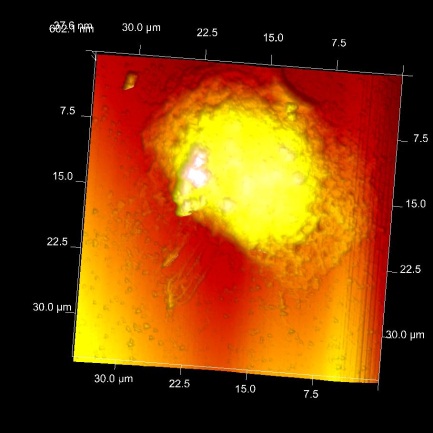

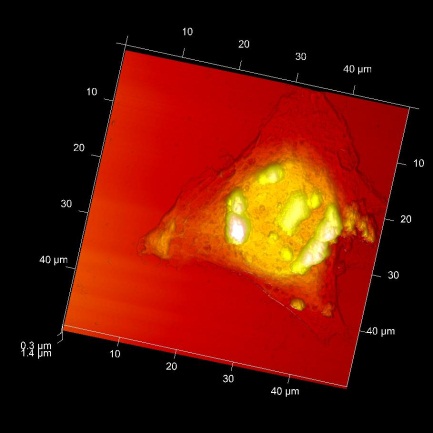


**A4**

**B4**

**C4**

Figure 1. AFM topography images of MCF-7 cells. A) control cells without MNPs, B) cells incubated with 25 µgmL^-1^ MNP-PEI and C) MNP-PEI-PEG over 1) 1 h, 2) 4 h, 3) 24 h and 4) 72 h. Cells were fixed after incubation and AFM imaging was performed in air using a RTESPA tip of spring constant 40 N/m, carrying out 896 scans/line at a scan rate of 0.32 Hz and 1.102 V amplitude.


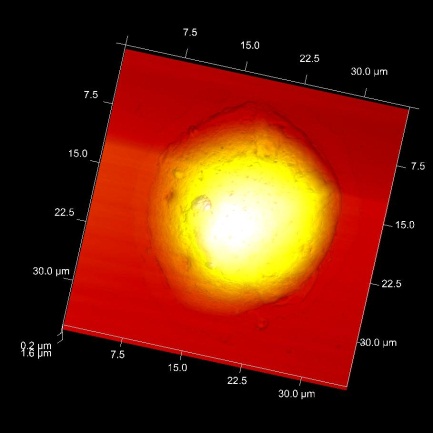
 **
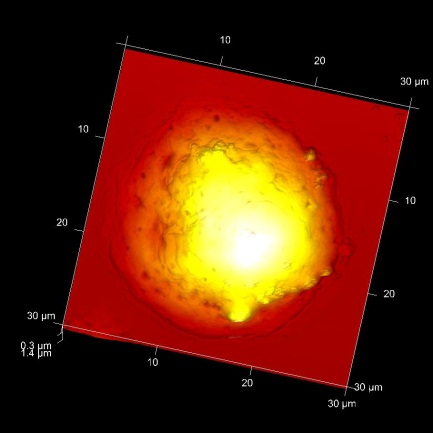
**
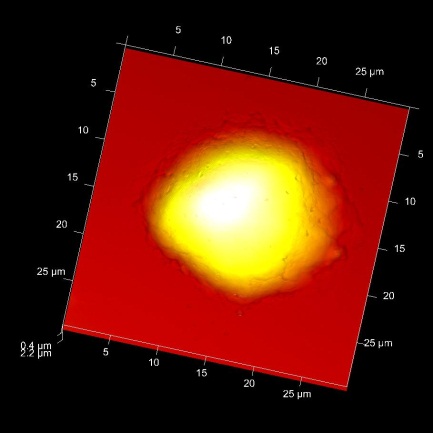


**A1**

**B1**

**C1**

**A2**

**
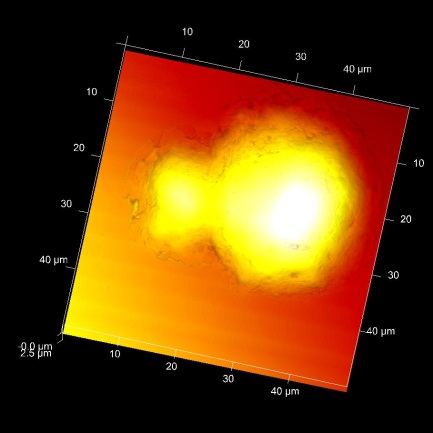

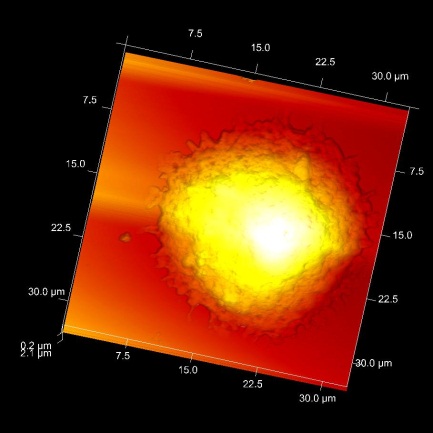

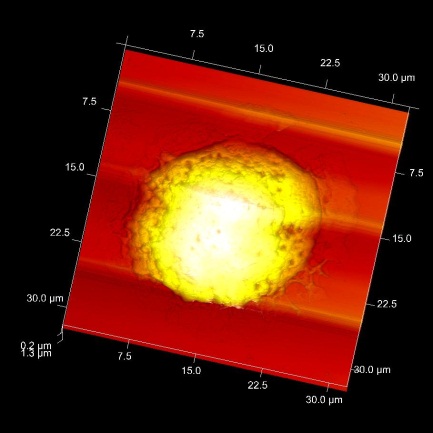
**

**A3**

**B3**

**C3**

**B2**

**C2**

**
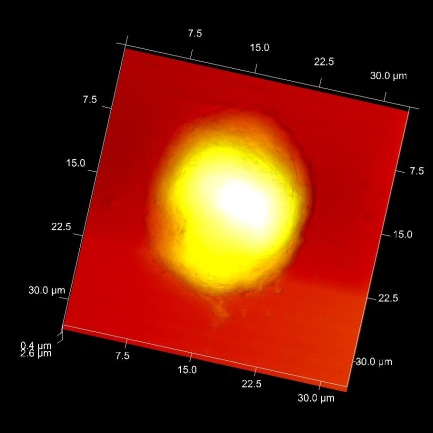

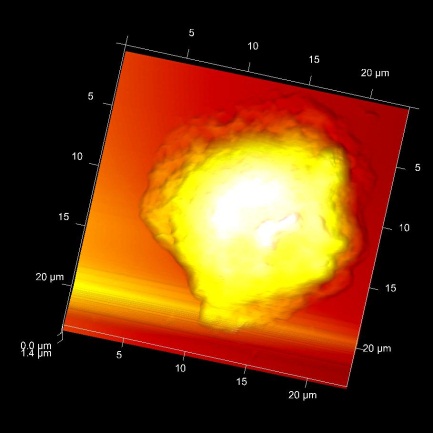

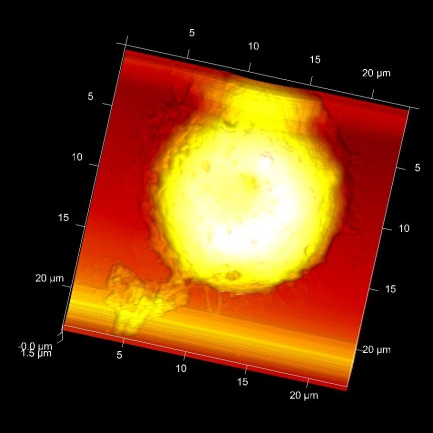
**

**B4**

**
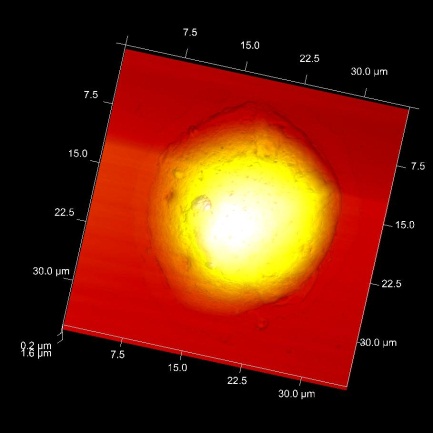

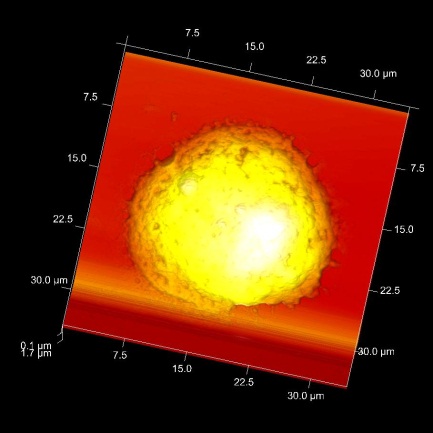

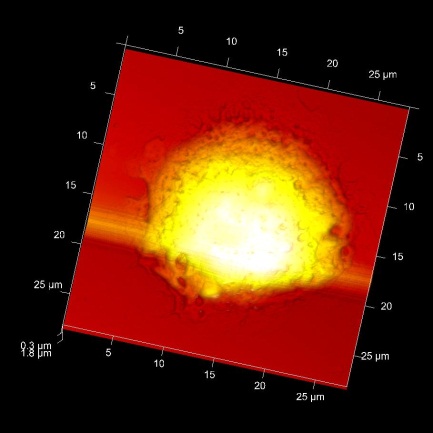
**

**A4**

**C4**

Figure 2. AFM topography images of U937 cells. A) control cells without MNPs, B) cells incubated with 25 µgmL^-1^ MNP-PEI and C) MNP-PEI-PEG over 1) 1 h, 2) 4 h, 3) 24 h and 4) 72 h. Cells were fixed after incubation and AFM imaging was performed in air using a RTESPA tip of spring constant 40 N/m, carrying out 896 scans/line at a scan rate of 0.32 Hz and 1.102 V amplitude.

Table 1. Percentage cytotoxicity on cell membrane measured via LDH leakage using MNP-PEI and MNP-PEI-PEG on MCF-7 cells over 1, 4, 24 and 72h (n=3 ±SD).

| Particle | Incubation time h | MNP concentration µgmL^-1^ | | | | | |
| --- | --- | --- | --- | --- | --- | --- | --- |
|  |  | 0 | 6.25 | 12.5 | 25 | 50 | 100 |
| MNP-PEI | 1 | 8.29 (0.040) | 8.02 (0.213) | 7.90 (0.048) | 7.87 (0.119) | 7.76 (0.025) | 7.86 (0.030) |
|  | 4 | 8.000 (0.11) | 8.28 (0.056) | 8.28 (0.152) | 8.32 (0.082) | 8.09 (0.218) | 8.26 (0.095) |
|  | 24 | 8.37 (0.187) | 10.60 (0.036)* | 10.65 (0.037)* | 10.54 (0.039)* | 10.47 (0.170)* | 10.94 (0.316)* |
|  | 72 | 8.19 (0.003) | 11.10 (0.173)* | 11.06 (0.238)* | 10.91 (0.038)* | 11.03 (0.059)* | 11.30 (0.044)* |
| MNP-PEI-PEG | 1 | 8.49 (0.092) | 8.82 (0.132) | 8.77 (0.076) | 8.73 (0.048) | 8.80 (0.072) | 8.73 (0.085) |
|  | 4 | 8.07 (0.067) | 8.53 (0.044) | 8.53 (0.050) | 8.44 (0.047) | 8.43 (0.052) | 8.45 (0.132) |
|  | 24 | 8.41 (0.080) | 8.35 (0.049) | 9.07 (0.633) | 8.04 (0.106) | 10.47 (0.159) | 8.23 (0.047) |
|  | 72 | 8.86 (0.014) | 9.60 (0.525) | 9.52 (0.700) | 9.38 (0.372) | 8.68 (0.074) | 8.89 (0.466) |

***** Denotes a significant increase from basal levels (p<0.05).

Table 2. Percentage cytotoxicity on cell membrane measured via LDH leakage using MNP-PEI and MNP-PEI-PEG on differentiated U937 cells over 1, 4, 24 and 72h (n=3 ±SD).

| Particle | Incubation time h | MNP concentration µgmL^-1^ | | | | | |
| --- | --- | --- | --- | --- | --- | --- | --- |
|  |  | 0 | 6.25 | 12.5 | 25 | 50 | 100 |
| MNP-PEI | 1 | 6.70 (0.028) | 6.54 (0.084) | 6.37 (0.118) | 6.20 (0.111) | 6.32 (0.102) | 6.18 (0.145) |
|  | 4 | 6.35 (0.066) | 6.58 (0.240) | 6.87 (0.587) | 6.57 (0.243) | 6.55 (0.242) | 6.37 (0.264) |
|  | 24 | 6.28 (0.231) | 5.31 (0.181) | 6.31 (0.989) | 6.12 (0.160) | 6.60 (0.161) | 6.32 (0.131) |
|  | 72 | 6.72 (0.000) | 6.04 (0.894) | 6.24 (0.890) | 6.00 (0.432) | 6.40 (0.892) | 6.24 (0.890) |
| MNP-PEI-PEG | 1 | 6.72 (0.000) | 6.57 (0.105) | 6.77 (0.111) | 6.20 (0.104) | 6.71 (0.103) | 6.44 (0.110) |
|  | 4 | 6.72 (0.000) | 6.44 (0.286) | 6.72 (0.292) | 6.82 (0.499) | 6.78 (0.292) | 6.44 (0.158) |
|  | 24 | 6.72 (0.000) | 6.24 (0.170) | 5.97 (0.145) | 5.98 (0.131) | 5.55 (0.140) | 6.31 (0.114) |
|  | 72 | 6.62 (0.100) | 6.01 (0.347) | 6.06 (0.007) | 6.85 (0.330) | 6.55 (0.195) | 6.45 (0.073) |

***** Denotes a significant increase from basal levels (p<0.05).

Table 3. ROS (% of control cells) and LPO induction by MNPs in MCF-7 cells incubated with 25 µgmL^-1^ for 1, 4, 24 and 72 h (n=3 ±SD).

| Particle | Incubation time h | ROS Assay LPO Assay | |
| --- | --- | --- | --- |
|  |  | % DCF fluorescence | MDA nM/mg protein  (Control cells: 4.351±0.365) |
| MNP-PEI | 1 | 107.67 (10.693) | 4.575 (0.510) |
|  | 4 | 133.67 (11.590)* | 4.618 (0.272) |
|  | 24 | 154.33 (12.503)* | 4.768 (0.488) |
|  | 72 | 128.67 (9.073)* | 4.648 (0.212) |
| MNP-PEI-PEG | 1 | 104.67 (2.517) | 3.901 (0.436) |
|  | 4 | 103.00 (2.000) | 3.833 (0.265) |
|  | 24 | 103.33 (4.163) | 4.670 (0.145) |
|  | 72 | 107.33 (4.041)* | 4.431 (0.265) |

***** Denotes a significant increase from basal levels (p<0.05).

Table 4. ROS (% of control cells) and LPO induction by MNPs in differentiated U937 cells incubated with 25 µgmL^-1^ for 1, 4, 24 and 72 h (n=3 ±SD).

| Particle | Incubation time h | ROS Assay LPO Assay | |
| --- | --- | --- | --- |
|  |  | % DCF fluorescence | MDA nM/mg protein  (Control cells: 1.116±0.105) |
| MNP-PEI | 1 | 105.67 (4.933) | 1.116 (0.009) |
|  | 4 | 101.67 (3.055) | 1.141 (0.030) |
|  | 24 | 110.33 (4.509)* | 1.204 (0.069) |
|  | 72 | 107.33 (6.807) | 1.116 (0.073) |
| MNP-PEI-PEG | 1 | 98.33 (1.155) | 1.153 (0.019) |
|  | 4 | 100.00 (1.732) | 1.112 (0.055) |
|  | 24 | 102.00 (2.656) | 1.087 (0.046) |
|  | 72 | 101.00 (1.000) | 1.082 (0.033) |

***** Denotes a significant increase from basal levels (p<0.05).
